# Supplementary material for: Validation of the Amharic version of perceived access to healthcare services for patients with cervical cancer in Ethiopia: A second-order confirmatory factor analysis
Source: PLoS One. 2024 May 15;19(5):e0300815. doi: 10.1371/journal.pone.0300815 (PMC11095753; doi:10.1371/journal.pone.0300815)
Supplement: S1 File — (DOCX) [file pone.0300815.s001.docx]

**Supporting information**

**Supporting table 1**: factor-specific reliability test results in R programming

| **Factor** | **Output** | **Improvement** |
| --- | --- | --- |
| Availability | Reliability analysis  Call: alpha(x = select(all308cfa, AV3, AV4))  raw_alpha std.alpha G6(smc) average_r S/N ase mean sd  0.76 0.76 0.62 0.62 3.3 0.027 3.6 0.77  median_r  0.62  95% confidence boundaries  lower alpha upper  Feldt 0.70 0.76 0.81  Duhachek 0.71 0.76 0.81  Reliability if an item is dropped:  raw_alpha std.alpha G6(smc) average_r S/N alpha se var.r  AV3 0.74 0.62 0.38 0.62 1.6 NA 0  AV4 0.52 0.62 0.38 0.62 1.6 NA 0  med.r  AV3 0.62  AV4 0.62  Item statistics  n raw.r std.r r.cor r.drop mean sd  AV3 308 0.92 0.9 0.71 0.62 3.5 0.93  AV4 308 0.88 0.9 0.71 0.62 3.8 0.78  Non missing response frequency for each item  1 2 3 4 5 miss  AV3 0.01 0.20 0.11 0.61 0.06 0  AV4 0.00 0.11 0.10 0.69 0.10 0 | The model has been improved after removing AV1 and AV2 making the alpha value 0.76 |
| Acceptability | Reliability analysis  Call: alpha(x = select(all308cfa, AC1, AC2, AC3, AC4, AC5, AC8, AC9))  raw_alpha std.alpha G6(smc) average_r S/N ase mean sd  0.93 0.93 0.93 0.66 14 0.0061 3.7 0.69  median_r  0.66  95% confidence boundaries  lower alpha upper  Feldt 0.92 0.93 0.94  Duhachek 0.92 0.93 0.94  Reliability if an item is dropped:  raw_alpha std.alpha G6(smc) average_r S/N alpha se var.r  AC1 0.92 0.92 0.91 0.66 11 0.0072 0.0035  AC2 0.92 0.92 0.91 0.66 12 0.0071 0.0032  AC3 0.91 0.91 0.90 0.64 11 0.0077 0.0021  AC4 0.93 0.93 0.92 0.68 13 0.0066 0.0013  AC5 0.92 0.92 0.91 0.66 12 0.0070 0.0027  AC8 0.92 0.92 0.91 0.66 12 0.0070 0.0028  AC9 0.92 0.92 0.91 0.66 12 0.0071 0.0034  med.r  AC1 0.66  AC2 0.67  AC3 0.63  AC4 0.68  AC5 0.68  AC8 0.65  AC9 0.66  Item statistics  n raw.r std.r r.cor r.drop mean sd  AC1 308 0.85 0.85 0.82 0.79 3.7 0.79  AC2 308 0.84 0.84 0.81 0.78 3.6 0.82  AC3 308 0.90 0.90 0.89 0.85 3.8 0.79  AC4 308 0.80 0.80 0.75 0.72 3.6 0.88  AC5 308 0.83 0.83 0.80 0.77 4.0 0.84  AC8 308 0.83 0.83 0.79 0.76 3.8 0.78  AC9 308 0.84 0.84 0.81 0.78 3.7 0.85  Non missing response frequency for each item  1 2 3 4 5 miss  AC1 0 0.13 0.12 0.68 0.07 0  AC2 0 0.16 0.11 0.67 0.06 0  AC3 0 0.12 0.09 0.69 0.09 0  AC4 0 0.18 0.11 0.63 0.08 0  AC5 0 0.08 0.08 0.55 0.28 0  AC8 0 0.10 0.10 0.68 0.12 0  AC9 0 0.15 0.11 0.64 0.09 0 | The model has been improved by dropping indicators AC6 and AC7 with final alpha value 0.92 |
| Affordability | Reliability analysis  Call: alpha(x = select(all308cfa, AF1, AF2, AF3))  raw_alpha std.alpha G6(smc) average_r S/N ase mean sd  0.85 0.87 0.86 0.7 6.9 0.016 3.9 0.88  median_r  0.6  95% confidence boundaries  lower alpha upper  Feldt 0.82 0.85 0.88  Duhachek 0.82 0.85 0.88  Reliability if an item is dropped:  raw_alpha std.alpha G6(smc) average_r S/N alpha se var.r  AF1 0.73 0.75 0.60 0.60 3.0 0.0286 NA  AF2 0.71 0.73 0.58 0.58 2.8 0.0306 NA  AF3 0.95 0.95 0.91 0.91 19.7 0.0055 NA  med.r  AF1 0.60  AF2 0.58  AF3 0.91  Item statistics  n raw.r std.r r.cor r.drop mean sd  AF1 308 0.90 0.93 0.93 0.80 3.7 0.89  AF2 308 0.91 0.94 0.94 0.82 3.7 0.87  AF3 308 0.85 0.81 0.62 0.60 4.1 1.20  Non missing response frequency for each item  1 2 3 4 5 miss  AF1 0.00 0.16 0.07 0.64 0.13 0  AF2 0.01 0.13 0.10 0.62 0.13 0  AF3 0.05 0.09 0.09 0.26 0.51 0 |  |
| Accommodability | Reliability analysis  Call: alpha(x = select(all308cfa, ACC1, ACC4, ACC5))  raw_alpha std.alpha G6(smc) average_r S/N ase mean sd  0.74 0.76 0.7 0.51 3.1 0.026 3.4 0.8  median_r  0.46  95% confidence boundaries  lower alpha upper  Feldt 0.69 0.74 0.79  Duhachek 0.69 0.74 0.80  Reliability if an item is dropped:  raw_alpha std.alpha G6(smc) average_r S/N alpha se var.r  ACC1 0.81 0.81 0.67 0.67 4.1 0.022 NA  ACC4 0.62 0.63 0.46 0.46 1.7 0.042 NA  ACC5 0.55 0.56 0.39 0.39 1.3 0.050 NA  med.r  ACC1 0.67  ACC4 0.46  ACC5 0.39  Item statistics  n raw.r std.r r.cor r.drop mean sd  ACC1 308 0.78 0.75 0.52 0.46 3.2 1.09  ACC4 308 0.82 0.84 0.74 0.61 3.5 0.92  ACC5 308 0.85 0.87 0.79 0.67 3.5 0.92  Non missing response frequency for each item  1 2 3 4 5 miss  ACC1 0.03 0.37 0.08 0.45 0.07 0  ACC4 0.01 0.24 0.08 0.63 0.04 0  ACC5 0.00 0.22 0.08 0.62 0.07 0 | Item ACC3 has been removed |
| Awareness | Reliability analysis  Call: alpha(x = select(all308cfa, AW1, AW2, AW3, AW4))  raw_alpha std.alpha G6(smc) average_r S/N ase mean sd  0.89 0.9 0.88 0.69 9.1 0.01 3.6 0.75  median_r  0.69  95% confidence boundaries  lower alpha upper  Feldt 0.87 0.89 0.91  Duhachek 0.87 0.89 0.91  Reliability if an item is dropped:  raw_alpha std.alpha G6(smc) average_r S/N alpha se var.r  AW1 0.84 0.86 0.82 0.66 5.9 0.0161 0.0153  AW2 0.91 0.91 0.88 0.77 10.3 0.0089 0.0015  AW3 0.84 0.85 0.80 0.65 5.7 0.0155 0.0062  AW4 0.86 0.87 0.83 0.69 6.6 0.0141 0.0086  med.r  AW1 0.61  AW2 0.79  AW3 0.66  AW4 0.66  Item statistics  n raw.r std.r r.cor r.drop mean sd  AW1 308 0.90 0.90 0.87 0.82 3.7 0.86  AW2 308 0.83 0.81 0.70 0.67 3.4 1.00  AW3 308 0.90 0.91 0.89 0.83 3.7 0.77  AW4 308 0.87 0.88 0.84 0.78 3.6 0.82  Non missing response frequency for each item  1 2 3 4 5 miss  AW1 0.00 0.16 0.09 0.65 0.09 0  AW2 0.01 0.26 0.09 0.56 0.08 0  AW3 0.00 0.12 0.10 0.70 0.07 0  AW4 0.00 0.16 0.10 0.68 0.06 0 |  |

**Supporting table 2**: Standardized regression weights (default model after bootstrapping) in Amos software

| **Parameter** | | | **Estimate** | **Lower** | **Upper** | **P** |
| --- | --- | --- | --- | --- | --- | --- |
| Accept | <--- | Perceived_access | .979 | .955 | 1.002 | .002 |
| Avail | <--- | Perceived_access | .922 | .857 | .982 | .002 |
| Accommod | <--- | Perceived_access | .823 | .753 | .888 | .002 |
| Aware | <--- | Perceived_access | .940 | .901 | .973 | .002 |
| Afford | <--- | Perceived_access | .815 | .733 | .886 | .003 |
| AC1 | <--- | Accept | .885 | .836 | .929 | .002 |
| AC2 | <--- | Accept | .811 | .723 | .879 | .003 |
| AC3 | <--- | Accept | .862 | .802 | .902 | .004 |
| AC4 | <--- | Accept | .721 | .639 | .782 | .003 |
| AC5 | <--- | Accept | .864 | .824 | .897 | .002 |
| AC8 | <--- | Accept | .778 | .677 | .846 | .004 |
| AC9 | <--- | Accept | .798 | .726 | .860 | .002 |
| AV3 | <--- | Avail | .647 | .553 | .735 | .003 |
| AV4 | <--- | Avail | .957 | .903 | 1.002 | .003 |
| AW1 | <--- | Aware | .853 | .797 | .906 | .002 |
| AW2 | <--- | Aware | .655 | .575 | .725 | .002 |
| AW3 | <--- | Aware | .933 | .900 | .962 | .002 |
| AF1 | <--- | Afford | .980 | .948 | 1.013 | .002 |
| AF2 | <--- | Afford | .926 | .876 | .959 | .003 |
| AF3 | <--- | Afford | .584 | .497 | .666 | .002 |
| ACC1 | <--- | Accommod | .522 | .434 | .610 | .002 |
| ACC4 | <--- | Accommod | .799 | .723 | .852 | .004 |
| ACC5 | <--- | Accommod | .842 | .763 | .905 | .003 |
| AW4 | <--- | Aware | .857 | .776 | .910 | .003 |

**Supporting table 3**: bias-corrected bootstrap standard errors in Amos software

| **Parameter** | | | **SE** | **SE-SE** | **Mean** | **Bias** | **SE-Bias** |
| --- | --- | --- | --- | --- | --- | --- | --- |
| Accept | <--- | Perceived_access | .000 | .000 | 1.000 | .000 | .000 |
| Avail | <--- | Perceived_access | .056 | .001 | .810 | .003 | .002 |
| Accommod | <--- | Perceived_access | .060 | .001 | .688 | .000 | .002 |
| Aware | <--- | Perceived_access | .045 | .001 | 1.004 | .001 | .001 |
| Afford | <--- | Perceived_access | .046 | .001 | 1.038 | .003 | .001 |
| AC1 | <--- | Accept | .000 | .000 | 1.000 | .000 | .000 |
| AC2 | <--- | Accept | .046 | .001 | .952 | .002 | .001 |
| AC3 | <--- | Accept | .046 | .001 | .975 | .003 | .001 |
| AC4 | <--- | Accept | .049 | .001 | .908 | .002 | .002 |
| AC5 | <--- | Accept | .051 | .001 | 1.038 | .002 | .002 |
| AC8 | <--- | Accept | .050 | .001 | .874 | .003 | .002 |
| AC9 | <--- | Accept | .044 | .001 | .967 | .002 | .001 |
| AV3 | <--- | Avail | .000 | .000 | 1.000 | .000 | .000 |
| AV4 | <--- | Avail | .079 | .002 | 1.247 | .004 | .002 |
| AW1 | <--- | Aware | .000 | .000 | 1.000 | .000 | .000 |
| AW2 | <--- | Aware | .038 | .001 | .892 | .000 | .001 |
| AW3 | <--- | Aware | .033 | .001 | .980 | .002 | .001 |
| AF1 | <--- | Afford | .000 | .000 | 1.000 | .000 | .000 |
| AF2 | <--- | Afford | .033 | .001 | .932 | .001 | .001 |
| AF3 | <--- | Afford | .070 | .002 | .806 | -.001 | .002 |
| ACC1 | <--- | Accommod | .000 | .000 | 1.000 | .000 | .000 |
| ACC4 | <--- | Accommod | .113 | .003 | 1.291 | .008 | .004 |
| ACC5 | <--- | Accommod | .116 | .003 | 1.365 | .008 | .004 |
| AW4 | <--- | Aware | .042 | .001 | .959 | .001 | .001 |
