# Supplementary material for: Validation of the Amharic version of perceived access to healthcare services for patients with cervical cancer in Ethiopia: A second-order confirmatory factor analysis
Source: PLoS One. 2024 May 15;19(5):e0300815. doi: 10.1371/journal.pone.0300815 (PMC11095753; doi:10.1371/journal.pone.0300815)
Supplement: S2 File — (DOCX) [file pone.0300815.s002.docx]

| **Dear Participant:**  Herewith, I will ask you to share me on your opinion regarding healthcare service delivery in cervical cancer, mainly of its accessibility. Your response will be to rate each statement as ’’absolutely disagree, disagree, neutral, agree or absolutely agree’’ depending your experience and perception. | | | | | | | |
| --- | --- | --- | --- | --- | --- | --- | --- |
| **Domains** | **Item constructs** | | **Responses** | | | | |
|  |  |  | 1  (absolutely disagree) | 2 (disagree) | 3 (neutral) | 4 (agree) | 5  (absolutely agree) |
| Availability | Q144 | There is adequate number of health professionals (doctors, nurses) to my need at the health facility. |  |  |  |  |  |
|  | Q145 | Health professionals (doctors, nurses…) have the appropriate skill to my healthcare needs. |  |  |  |  |  |
| Acceptability | Q146 | The quality of services provided in the health facility is acceptable. |  |  |  |  |  |
|  | Q147 | The health facility staff meets the needs of the clients in various ways, such as education, care and support. |  |  |  |  |  |
|  | Q148 | Health workers listen carefully to what I have to say. |  |  |  |  |  |
|  | Q149 | The health workers give me enough time. |  |  |  |  |  |
|  | Q150 | I trust the statements of the treatment team (doctor, nurse, etc.) about my health and illness. |  |  |  |  |  |
|  | Q153 | The treatment team at the health facility is respectful. |  |  |  |  |  |
|  | Q154 | The health workers (doctors, nurses etc.) are familiar with the culture and religion of clients and communicate with them appropriately. |  |  |  |  |  |
| Affordability | Q155 | To solve my health problem, I first see a general practitioner. |  |  |  |  |  |
|  | Q156 | With the guidance of a general practitioner, I use specialized and sub-specialized services. |  |  |  |  |  |
|  | Q157 | Cost is a serious barrier to using health care. |  |  |  |  |  |
| Accommodation | Q158 | It is easy to make an appointment at a health facility. |  |  |  |  |  |
|  | Q161 | The working hours of the health facility are suitable for receiving healthcare services. |  |  |  |  |  |
|  | Q162 | The physical space of the health facility is suitable for receiving services. |  |  |  |  |  |
| Awareness | Q164 | The education that is given to me is such that I understand them. |  |  |  |  |  |
|  | Q165 | The information I need is expressed in simple language without the use of specialized words. |  |  |  |  |  |
|  | Q166 | Communication of health workers (doctor, nurse etc.) with clients is appropriate. |  |  |  |  |  |
|  | Q167 | Health workers try to make sure I fully understand the health information provided. |  |  |  |  |  |
