# Supplementary material for: Validation of the Amharic version of perceived access to healthcare services for patients with cervical cancer in Ethiopia: A second-order confirmatory factor analysis
Source: PLoS One. 2024 May 15;19(5):e0300815. doi: 10.1371/journal.pone.0300815 (PMC11095753; doi:10.1371/journal.pone.0300815)
Supplement: S3 File — (DOCX) [file pone.0300815.s003.docx]

| ውድ ታካሚ፡  ከዚህ በመቀጠል የማህጸን ጫፍ ካንሰር ህክምና አገልግሎት አሰጣጥ፤ በተለይም አቅርቦትና ተደራሽነት በተመለከተ እይታዎን እንዳጋሩኝ አንዳንድ ጥያቄዎችን አነብልዎታለሁ፡፡ የእርስዎ ምላሽ ምን ያህል እንደሚሰማሙ ማለትም፤ “በጣም አልስማማም”፤ “አልስማማም”፤ “ልዩነት የለኝም”፤ “እስማማለሁ” ወይም “በጣም እስማማለሁ” በማለት ይሆናል፡፡ | | | | | | | |
| --- | --- | --- | --- | --- | --- | --- | --- |
| ወካይ መለኪያ | መጠይቆች | | ምላሽ | | | | |
|  |  |  | 1  በጣም አልስማማም | 2 አልስማማም | 3  ልዩነት የለኝም | 4 እስማማለሁ | 5  በጣም እስማማለሁ |
| የአገልግሎቱ ወይም መድሃኒቱ መገኘት | ጥ145 | የተቋሙ ጤና ባለሙያች (ሀኪም፤ ነርስ ወዘተ) ቁጥርና ስብጥር ለምፈልገው የጤና አገልግሎት በቂ እና ምቹ ነው፡፡ |  |  |  |  |  |
|  | ጥ146 | የጤና ባለሙያዎቹ (ሀኪም፤ ነርስ ወዘተ ) በምፈልገው አገልግሎት ላይ በቂ የሙያ ክህሎት አላቸው፡፡ |  |  |  |  |  |
| ቅቡልነት | ጥ147 | በተቋሙ የሚሰጠው አገልግሎት ጥራቱን የጠበቀ ነው፡፡ |  |  |  |  |  |
|  | ጥ148 | የተቋሙ ሙያተኞች የታካሚዎችን ፍላጎት በተለያዬ መልኩ፤ለምሳሌ ትምህርት፤ እንክብካቤ እና እርዳታ በመስጠት የሚያሟሉ ናቸው፡፡ |  |  |  |  |  |
|  | ጥ149 | ጤና ባለሙያዎች እኔም የማነሳውን ሀሳብ በአግባቡ ያዳምጡኛል፡፡ |  |  |  |  |  |
|  | ጥ150 | የጤና ባለሞያዎቹ በቂ ጊዜ ይሰጡኛል፡፡ |  |  |  |  |  |
|  | ጥ151 | የጤና ባለሙያ ቡድኑ (ሀኪም፤ ነርስ፤ ወዘተ) ስለጤናም ሆነ ህመም ሁኔታዬ ለሚሰጡኝ ምክር እምነት አለኝ፡፡ |  |  |  |  |  |
|  | ጥ154 | የህክምና ቡድኑ ለታካሚዎች አክብሮት አላቸው፡፡ |  |  |  |  |  |
|  | ጥ155 | ጤና ባለሙያዎቹ (ሀኪሞች፤ ነርሶች፤ ወዘተ) የታካሚዎችን ባህል እና እምነት የሚያውቁና በተገቢው መንገድ የሚያሰተናግዱ ናቸው፡፡ |  |  |  |  |  |
| የመክፈል አቅምን ያገናዘበ መሆን | ጥ156 | የጤና ችግሬን ለመፍታት መጀመሪያ በጠቅላላ ሀኪም እታያለሁ፡፡ |  |  |  |  |  |
|  | ጥ157 | በጠቅላላ ሀኪም ምክር መሰረት የስፔሻሊስት ወይም ሰብ ስፔሻሊስት ሀኪም አገልግሎት እጠቀማለሁ፡፡ |  |  |  |  |  |
|  | ጥ158 | የጤና አገልግሎት ለማግኘት ወጪ ትልቅ እንቅፋት ሆኖብኛል፡፡ |  |  |  |  |  |
| አካታችነት | ጥ159 | በጤና ተቋሙ ቀጠሮ ለመያዝ ቀላል ነው፡፡ |  |  |  |  |  |
|  | ጥ162 | የጤና ተቋሙ የስራ ሰዓት ሁሉንም አገልግሎቶች ለማግኘት ምቹ ነው፡፡ |  |  |  |  |  |
|  | ጥ163 | የተቋሙ ግቢ የሚሰጡትን አገልገሎቶች ለማግኘት ምቹ ነው፡፡ |  |  |  |  |  |
| ለግንዛቤ የሚቀል | ጥ165 | የጤና ትምህርት ሲሰጠኝ እኔ በቀላሉ በምረዳው መልኩ ተደርጎ ነው፡፡ |  |  |  |  |  |
|  | ጥ166 | የሚሰጠኝ መረጃ ቀላል በሆነ ቋንቋ እና ለመረዳት የሚያስቸግሩ ቃላት የሌሉበት ነው፡፡ |  |  |  |  |  |
|  | ጥ167 | የጤና ባለሙያዎቹ (ሀኪም፤ ነርስ፤ ወዘተ) ከታካሚ ጋር ያላቸው ተግባቦት ጥሩ ነው፡፡ |  |  |  |  |  |
|  | ጥ168 | የጤና ባለሙያዎች የተሰጠኝን የጤና መረጃ በአግባቡ ስለመረዳቴ ለማረጋገጥ ይጥራሉ፡፡ |  |  |  |  |  |
