# Supplementary material for: Validation of the Amharic version of perceived access to healthcare services for patients with cervical cancer in Ethiopia: A second-order confirmatory factor analysis
Source: PLoS One. 2024 May 15;19(5):e0300815. doi: 10.1371/journal.pone.0300815 (PMC11095753; doi:10.1371/journal.pone.0300815)
Supplement: Attachment — Submitted filename: response to reviewers comments.docx [file pone.0300815.s004.docx]

| **SN** | **Response to reviewers’ comments**  Dear editor and reviewers,  We are pleased to inform you that we have successfully addressed the comments and concerns raised during the review process. We express our sincere gratitude for the time and insightful comments you dedicated to the thorough evaluation of our manuscript. Your constructive feedback has played a crucial role in enhancing the quality and clarity of our work. Each comment was carefully considered, and the necessary revisions were implemented to strengthen the overall coherence and robustness of the manuscript. Your expertise and commitment to scholarly excellence have been instrumental in shaping our work into a more comprehensive and impactful contribution to the field. Kindly, note that the changes made according to each comment have been inserted in the appropriate place and highlighted in red color. This version has been renamed as ‘manuscript with highlight’. Separately, we have also submitted a revised file without highlight as ‘manuscript without highlight’. If you have more concerns, please let us know.  With kind regards,  Tariku Shimels, corresponding author | |
| --- | --- | --- |
|  | **Reviewer 1 comments** | **Responses** |
| 1 | Abstract-Be more specific about the objectives of your study. Clearly state how validating the Amharic-version of the instrument contributes to the field. | Thank you for your valuable input. Considering your comments and mindful of the abstract word limit, we have added a precise justification on the contribution of validating a local version of those tools. (please see page 1) |
| 2 | Abstract- Provide more details on the consecutive sampling method. Clarify how it ensures a representative sample of the population. | Thank you for the feedback on this. The suggestion has been accepted an included in the methods section as adding further explanation of the abstract may exceed the journal’s word limit. (please check page 4 ) |
| 3 | Abstract - Provide more in-depth interpretation of your results. Discuss the implications of the internal consistency and validity tests. | Thank you for the comment. The suggestion has been accepted. A rephrase statement has been added as ‘The study indicated a high internal consistency and validity of items with good fit to the data, suggesting potential accuracy of the domains.’ (please refer page 1 ) |
| 4 | Introduction-Clarify and distinguish between the various definitions and dimensions of access to healthcare. Consider using a table or a diagram to visually represent the different models and dimensions proposed by various authors. | Thank you for the comment. We have accepted your suggestion and included a table showing this. (Please see page 3). |
| 5 | Provide more context or examples to illustrate the differences between the dimensions of access, such as "affordability," "availability," "accessibility," etc. | Thank you for the comment. A detail of this has been addressed accordingly. (see page 3&4) |
| 6 | Strengthen the link between the various theories and the context of your study. Explicitly mention how these theories are relevant to the Ethiopian context. | Thank you for the comment. A link between the theories of interest, especially, in the developing context and the study’s context has been shown in the last section of third paragraph. (See page 4). |
| 7 | Include a brief discussion on how the understanding of access to healthcare has evolved over time, and what gaps your study aims to fill. | Thank you for the comment. We have included statements on the development of literatures on access in recent times, but how there is a gap in the existing methods, and why this study would fill that gap. (see page 4) |
| 8 | Emphasize the relevance of the study to the Ethiopian context early in the section. Discuss the specific challenges related to healthcare access in Ethiopia and how they relate to the models and dimensions of access you've described | Thank you for the comment. Rational on the relevance of doing this study to the Ethiopian context, specifically, in patients with cervical cancer has been presented. (see page 4) |
| 9 | Provide statistics or recent studies highlighting the healthcare access situation in Ethiopia, especially in relation to cervical cancer care. | Thank you. We have added a couple of recent literature directly related to the question. (please check page 3) |
| 10 | Discuss the limitations of existing methods to measure patient perceptions of healthcare access. Highlight why these may be inadequate or less applicable in the Ethiopian context, paving the way for the need for your study. | Thank you for the comment. We accepted your suggestion and incorporated the same section. Please find the detailed edits on page 4 and 5. |
| 11 | Mention any specific shortcomings in previous studies that used these methods in similar contexts, and how your study addresses these shortcomings. | Thank you for the comment. The shortcoming to previous studies is particularly with regard to using non-validated tools, working on only fragmented tools, or use of qualitative methods. Generally, the unavailability of a validated and Amharic version in the country was a gap which this study attempts to fill. This has been reiterated on page 4 and 5. |
| 12 | Ensure the section flows logically from the general understanding of healthcare access to the specifics of the Ethiopian context. | Thank you. We have made arrangements in the introduction section to keep the logical flow of paragraphs, including along with previous paragraphs (the table with summary of the literature. Please see pages 2 to 5. |
| 13 | Avoid jargon or overly technical language to make the section accessible to readers from various backgrounds. | Thank you for the concern. We have tried to avoid unclear or jargons in the section. |
| 14 | Expand the description of the oncology centers in Addis Ababa to provide context for readers unfamiliar with the Ethiopian healthcare system. This might include the capacity of these centers, typical patient demographics, etc. | Thank you for the comment. An extensive description of the study sites has been provided under the ‘study context, design and population’ subsection. (see page 5) |
| 15 | Explain why a cross-sectional design is appropriate for this study and how it helps in achieving the study objectives. | Thank you for the comment. A description stating on the advantage on the suitability of the proposed study design has been supplied per your request. (see page 6) |
| 16 | Provide more detailed reasoning for choosing the specific validity and reliability tests used. This includes why these tests are appropriate for your study population and the instrument being validated. | Thank you for the comment. In addition to the earlier guidelines followed, an explanation on why validity & reliability should be done has been provided under the subsection ‘validity and reliability of the instrument.’ (see page 6) |
| 17 | Discuss any potential limitations of these methods and how they might affect the interpretation of your results. | Thank you for the comment. We have added a description indicating that there could be difficulty in ensuring both validity and reliability of all items given the intricate nature of the construct measured in this study. (see page 6) |
| 17 | Clarify how the psychometric domains and constructs were specifically adapted from Hoseini et al.'s questionnaire to fit the Ethiopian context and the specific needs of cervical cancer patients. | Thank you for this comment. It has also been raised by the reviewer 2 and the following explanation was added. We chose to adopt the tool developed by Hoseini and colleagues because it represents the most recent iteration among related tools in the field. Moreover, this particular tool has undergone testing and validation in a context and health system highly akin to ours. (please see page 6) |
| 18 | Explain the rationale behind each modification made to the original instrument. | Thank you for this question. Kindly note that all reasons to modify the original too have been presented in detail under the section ‘instrument design and adaptation’ on page 7. |
| 19 | Detail the qualifications and expertise of the translators to ensure their competency in translating a psychometric instrument. | Thank you for the comment. We have added details of the translators as shown on page. (see page 7) |
| 20 | Discuss how you addressed any cultural nuances in translation that might affect the interpretation of the questions. | Thank you for your comment. We have accepted your comments and have mentioned the several steps used to address the cultural nuances in translation. The edits to this point have been added under the section ‘instrument design and adaptation’. (see page 7). |
| 21 | Elaborate on how the panel of experts was selected, including their expertise and relevance to the study. | Thank you. We added on the selection and profession/expertise of the panels involved. Please see revision made on page 8. |
| 22 | Explain how the feedback from the panel was incorporated into the final instrument. | Thank you for rising on this. The systematic approach used during the feedback incorporation process has been added per your comments. (see page 8) |
| 23 | Describe the characteristics of the pilot sample and how they compare to the main study population. | Thank you for the comment. A revision concerning this question has been added under the subsection ‘piloting of the instrument’. (see page 10 ) |
| 24 | Justify the use of Cronbach's alpha and confirmatory factor analysis in the context of your study. Discuss how these analyses contribute to the overall validation of the instrument. | Thank you for the comment. This question has been addressed in detail on page 10. please see text highlighted. |
| 25 | Explain the training provided to data collectors and how it contributes to the quality and consistency of the data collected. | Thank you for the comment. A revision has been included under the section ‘data collection process and quality assurance. Please on page 12. |
| 26 | Describe any quality control measures taken during data collection. | Thank you for the question. This question has been addressed on the same section to above (see page 12). |
| 27 | Elaborate on the choice of statistical methods and software used for data analysis. | Thank you for the question. The chosen statistical methods depend on the data collection methods used, and the type of reports required. As the data was collected in KoboCollect tool, data was exported to MS-Excel, and then descriptive results were generated in R software, and optionally in SPSS. As the annexed results are of R output we ignored mentioning the SPSS-because it has no additional merit. The Amos software was found best to generate SEM plots. At the same time, the models of analysis chosen depend on the nature of data and the assumptions for the particular model. Test of normality of the aggregate score and equality of variance were the common assumptions tested and determined the specific methods used. Please note that we did not include edits regarding this in manuscript, and let’s know if we should do. |
| 28 | Discuss how these methods are suitable for the type of data collected and the research questions posed | Thank you also for raising this point. As we stated in Q27 above, the methods and models used are appropriate to the data collected and answer specific aspects of the research question. A statement addressing this issue has been placed at the end of the section ‘data analysis’. (see page 12) |
| 29 | Explain the process of obtaining informed consent and how the study ensures confidentiality and privacy of the participants | Thank you for the concern. The process of obtaining informed consent has been provided under the section ‘ethics consideration’. Shortly, the necessary steps of informed consent process has been obtained through explaining the study objective, informing the benefit and potential risks (time that can be consumed and fatigue), and answering questions that arise. A detail has been added to the last section of the ethics consideration section.(see page 13) |
| 30 | Provide a brief interpretation of the participant characteristics, highlighting any notable findings such as the high number of referred patients from outside Addis Ababa or the demographic trends observed. | Thank you for the comment. We have added an interpretation of the findings for the section ‘participant characteristics’. (see highlighted text on page 13). |
| 31 | Consider discussing the implications of these characteristics on the generalizability of the study findings. | Thank you. This question has been addressed in the same section to above. The implication is that the tool can be applicable to broader patient demographics obtained in the study. |
| 32 | Elaborate on the rationale for modifying specific components of the instrument, such as changing 'access' to 'reachability' and the inclusion of different cancer types. | Thank you for he concern and comment. We have a further explanation why the modifications were made. (please see on page 15) |
| 33 | Discuss the implications of the content validity ratio (CVR) and item-level content validity index (I-CVI) values obtained. Explain what these values suggest about the quality of your instrument. | Thank you for the comment. An interpretation has been added to the section ‘initial validity and reliability assessment’. (see page 15) |
| 34 | Provide an interpretation of the Cronbach’s alpha values obtained for each factor. Discuss what these values indicate about the reliability of the instrument. | Thank you for the comment again. An interpretation has been added to the section ‘initial validity and reliability assessment, specific to the implication of obtained cronbach’s alpha result’. (see page 15) |
| 35 | Clarify the decision-making process behind the removal of specific items from the factors based on the reliability analysis. | Thank you for the question. As described in the section, the decision in the removal of an item was guided by the results of the reliability test and factor loading scored. According to several sources, an item with a Cronbach’s alpha <0.7 and a factor loading less than 0.5 poorly fits to be included in the construct. Kindly note that we did not add other than what is presented as we assume it has been adequately described. |
| 36 | Interpret the composite reliability (CR) and average variance extracted (AVE) for each factor. Explain how these values confirm the convergent validity of the instrument. | Thank you for the comment. An interpretation to the result obtained has been provided. The fact that CR and AVE scored 0.6 or more suggested good fit implying that the data fits the construct. (see on page 17) |
| 37 | Discuss any factors that did not meet the recommended thresholds and what this might imply. | Thank you. But, all factors have met the threshold level for the CR test at this level. |
| 38 | Explain the significance of the hetero-trait to mono-trait (HTMT2) ratio results. Discuss the implications of the two factor pairs that showed higher values than the recommended threshold. | Thank you. The brief significance of HTMT2 result as well as the implication of slightly higher value of the two pairs has been presented in the section. (see page 17) |
| 39 | Interpret the findings from the known group validity analysis. Discuss how the instrument differentiates between groups based on income, health insurance status, social support, and occupation. | Thank you for the comment. WE have added interpretation to the results of the known group validity. Please find results highlighted on page 18. |
| 40 | Explain the significance of these findings in the context of assessing perceived access to healthcare. | Thank you for the comment. We have added implication of the above results in terms of assessing access to healthcare. Please find results highlighted on page 18. |
| 41 | Provide a detailed interpretation of the model fitness indices. Discuss what each index (e.g., CFI, TLI, SRMR, RMSEA) indicates about the overall fit of your model. | Thank you. A summarized paragraph interpreting the results has been presented under the section ‘model fitness indices’. Please see details highlighted on page 19. |
| 42 | Reiterate the study's primary aim at the beginning of the discussion to remind readers of the context. Link back to how your findings fulfill this aim, emphasizing the significance of validating the Amharic version of the psychometric measure in the Ethiopian context. | Thank you for the comment. A detail on the aim and implications of the Amharic version of the tool has been provided per your question. (please see on page 21) |
| 43 | Discuss the implications of the high internal consistency and the decision to drop certain items in more detail. Explore how these adjustments improve the instrument's applicability and reliability. | Thank you for the comment. Please find the implications of the high internal consistency result as well as the basis for decision to drop items, and the resulting improvement on the model’s applicability. (see page 22) |
| 44 | Explain the significance of the convergent, divergent, and known-group validity findings in relation to the quality and utility of the instrument. | Thank you for the comment. The details on the implication and significance of each validity component has been presented on page 23. |
| 45 | Delve deeper into the multicollinearity observed between certain constructs. Discuss potential reasons for this overlap and how it might affect the interpretation of results. | Thank you. The issue has been addressed by emphasizing on the constructs, the possible reasons and how it might affect results measured using the tool. (see page 23) |
| 46 | Consider whether this finding suggests a need for further refinement of the instrument or reflects the interconnected nature of these constructs in the context of healthcare access. | Thank you. The possible reason of connection between the two pairs of constructs has been suggested per your comment. A detail has been shown on page 23. |
| 47 | Provide a more nuanced interpretation of the model fitness indices, discussing how each index contributes to the overall validation of the instrument. | Thank you for the comment. A detailed explanation has been presented in the discussion section per your suggestion. please see highlighted text on page 24. |
| 48 | Address the moderate fit indicated by RMSEA and what it might imply about the model's applicability. | Thank you again. This issue has been addressed in the question/comment under 47. (see page 24) |
| 49 | Compare and contrast your findings with Elaborate on each limitation mentioned. For instance, discuss how the sample size might have impacted the findings and what could be done in future studies to mitigate this. | Thank you. A description on the implication of the limitations stated had been made. The recommendation section has been added to the end of the conclusion section. (see on page 24) |
| 50 | Address the potential impact of relying on constructs from previous literature and how this might have influenced the outcomes. | Thank you. A description on the implication of using a constructs from a previous study has been stated. (see on page 24) |
|  | **Reviewer 2 comments** | **Responses** |
| 1 | Firstly, the paper must be intensively revised considering the English language and the grammatical in some parts. | Thank you for the comment. We have now edited the manuscript for grammatical and language errors throughout the manuscript. |
| 2 | Then, it could be better to explain the reason why, for the study, the Authors decided to use the questionnaire provided in the literature by Hoseini and colleagues. | Thank you for your inquiry. We chose to adopt the tool developed by Hoseini and colleagues because it represents the most recent iteration among related tools in the field. Moreover, this particular tool has undergone testing and validation in a context and health system highly akin to ours. To reinforce this assertion, please refer to the highlighted text on page 6, where we have incorporated an additional statement supporting this rationale. |
| 3 | …it could be better to describe in a more appropriate way the experts' panel involved, also dividing the methodological aspects and the used materials from the obtained results and the calculated statistical indexes. | Thank you for the comment. The reason why the broad mix of expert panels has been described following your comments (please see page 6). While the phases of the qualitative and quantitative methods were performed for specific reasons as stated in the methods section, the changes have been described in the results section, such as ‘reachability’ and ‘colon or breast cancer’ instead of ‘cervical cancer’ in the qualitative section before proceeding to the quantitative evaluations. |
| 4 | The last comment is related to the anonimity and the ethical concerns of the study. In the methodology it is defined that the participants provided a verbal informed consent, but a written informed consent was not provided? | Thank you for the concern on the ethics aspect in this study. There are two reasons why we did not consider taking written informed consent. One is that there is less than the usual minimal risk expected that the participants would encounter as a result of being included in the study, rather the benefits outweighs the discomforts that possibly arise. As can be observed from the procedures of the study and the findings documented, this has been safeguarded throughout and after the completion of the study. Secondly, people in the study context generally have anticipation that when asked for a signature, there may be some liability or risk that may follow. People may speculate on the possible consequences of taking part, and may decide to drop out of the study, filled by unnecessary fear, while actually no risk exists. The latter is a minimal reason and does not violate participants’ voluntary participation. That is why the ethics committee approved the protocol.  In addition, the necessary steps of informed consent process has been obtained through explaining the study objective, informing the benefit and potential risks (time that can be consumed and fatigue), and answering questions that arise. A detail has been added to the last section of the ethics consideration section. (see page 13) |
