# Supplementary material for: Validation of the Amharic version of perceived access to healthcare services for patients with cervical cancer in Ethiopia: A second-order confirmatory factor analysis
Source: PLoS One. 2024 May 15;19(5):e0300815. doi: 10.1371/journal.pone.0300815 (PMC11095753; doi:10.1371/journal.pone.0300815)
Supplement: Attachment — Submitted filename: Response to the editor or reviewer 2.docx [file pone.0300815.s005.docx]

| **Dear Esteemed Editor,**  We extend our sincere gratitude to you for the invaluable comments and suggestions you provided. Your insights have been truly enlightening and have significantly enhanced the quality of the manuscript. Your expertise and attention to detail have made a substantial impact on the clarity and coherence of the content. We appreciate the time and effort you invested in reviewing the manuscript thoroughly. Your constructive feedback has not only improved the overall structure but has also contributed to a more comprehensive and robust presentation of the research findings.  Once again, thank you for your dedication to ensuring the excellence of this work. We hope that we have addressed all your comments and suggestions. Please let us know if you have more enquiries.  Kindest regards,  Tariku S, corresponding author | | |
| --- | --- | --- |
| **S.no** | **Editor comments** | **Responses** |
| 1 | Provide a bit more context regarding the Ethiopian healthcare system and any unique challenges it faces. This will help readers unfamiliar with the Ethiopian context to better understand the significance of the study. | Thank you for the insightful comment. We have included a further detail about the Ethiopian health system, the national plan on cancer control and the status of cancer, especially, cervical cancer despite the efforts. Kindly find the edits on page 5. |
| 2 | To enhance the quality of your introduction, consider incorporating the following references   1. <https://link.springer.com/article/10.1007/s12144-021-01662-2> 2. <https://pubmed.ncbi.nlm.nih.gov/35943005/> 3. <https://www.archbreastcancer.com/index.php/abc/article/view/470> | Thank you for the suggestion. We have now included the recommended sources in the introduction section. Please see the highlighted text on page 4. |
| 3 | Ensure that terminology is consistently defined. For instance, the term "psychometric component" could be briefly explained for readers who may not be familiar with it. | Thank you for the comment. An extended explanation has been added following your concern. Kindly find the added edits at the end of the introduction on page 5. |
| 4 | Provide more detailed explanations of the steps involved in the validation process. For instance, elaborate on the qualitative and quantitative approaches used for content validity and explain the significance of each step | Thank you for the comment. We have stated the main activities performed in the validation process as design and adaptation (phase I), pilot testing (phase II), and factorial analysis (phase III) at the beginning of paragraph 2 in the discussion. We have then extended the discussion on the process and significance of the qualitative and quantitative methods used. Please see highlighted text on page 21. |
| 5 | Emphasize the importance of future research efforts focusing on larger sample sizes, refinement of measurement items, and the adoption of a more context-specific approach to construct definition. | Thank you for the concern and suggestion. While the limitations of the study raised those issues at the end of the discussion section, a recommendation on the same has been added at the end of the conclusion section. Kindly check the highlighted text on pages 21 &22. |
